# New Bioactive Alkyl Sulfates from Mediterranean Tunicates

**DOI:** 10.3390/molecules171112642

**Published:** 2012-10-26

**Authors:** Concetta Imperatore, Anna Aiello, Filomena D’Aniello, Paolo Luciano, Rocco Vitalone, Rosaria Meli, Giuseppina Mattace Raso, Marialuisa Menna

**Affiliations:** 1 The NeaNat Group, Dipartimento di Chimica delle Sostanze Naturali, Università degli Studi di Napoli “Federico II”, Via D. Montesano 49, 80131, Napoli, Italy; Email: cimperat@unina.it (C.I.); aiello@unina.it (A.A.); filomena.daniello@unina.it (F.D.); rocco.vitalone@unina.it (R.V.); 2 C.S.I.A.S. (Centro Servizi Interdipartimentale di Analisi Strumentale), Università degli Studi di Napoli “Federico II”, Via D. Montesano 49, 80131, Napoli, Italy; Email: pluciano@unina.it; 3 Dipartimento di Farmacologia Sperimentale, Università degli Studi di Napoli “Federico II”, Via D. Montesano 49, 80131, Napoli, Italy; Email: rosaria.meli@unina.it (R.M.); giuseppina.mattaceraso@unina.it (G.M.R.)

**Keywords:** natural products, alkyl sulfate, cytotoxic activity, structure elucidation, ascidians

## Abstract

Chemical investigation of two species of marine ascidians, *Aplidium elegans* and *Ciona edwardsii*, collected in Mediterranean area, led to isolation of a series of alkyl sulfates (compounds **1**–**5**) including three new molecules **1**–**3**. Structures of the new metabolites have been elucidated by spectroscopic analysis. Based on previously reported cytotoxic activity of these type of molecules, compounds **1**–**3** have been tested for their effects on the growth of two cell lines, J774A.1 (BALB/c murine macrophages) and C6 (rat glioma) *in vitro*. Compounds **1** and **2** induced selective concentration-dependent mortality on J774A.1 cells.

## 1. Introduction

**Figure 1:**
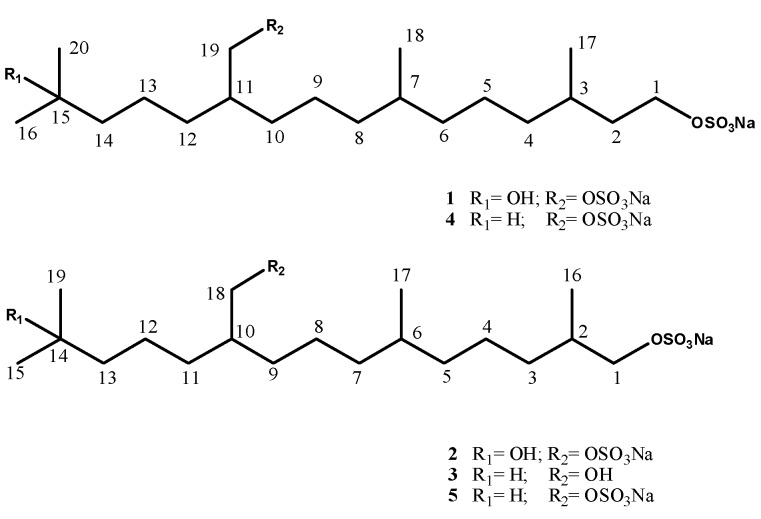
Structures of compounds **1**–**5**.

Ascidians have been an extremely rich source of sulfur-containing molecules which, on the other hand, are quite unusual in marine organisms. A number of sulfides/polysulfides, sulphur heterocycles, sulfoxides, and alkyl sulfates have been isolated from marine ascidians [1,2]. In particular, these latter compounds, although occasionally reported from marine source [3,4,5,6], have shown to be often present in remarkable amounts in solitary ascidians of the families Ascididae and Pyuridae, as well in colonial Polyclinidae species [7,8,9,10,11,12,13,14]. They often have quite simple structures, mostly of polyketide derivation even though in some frameworks an isoprenoid origin is clearly recognized; almost all the isolated compounds are endowed with cytotoxic and/or antiproliferative activity. In the course of our program on discovery of cytotoxic metabolites from Mediterranean ascidians [15,16], we have analyzed the chemical composition of methanol extracts of two species, *Aplidium elegans* (Monniot & Monniot, 1983) and *Ciona edwardsii* (Roule, 1884), both collected in the Bay of Naples, Italy. This investigation yielded, in addition to the known sulfated terpenoids **4** and **5** [10,14], some analogues, compounds **1** and **2** from *A. elegans*, and compound **3** from *C. edwardsii* (Figure 1). The present paper deals with the isolation and structure elucidation of the three new compounds as well as with their cytotoxic activity measured *in vitro* on J774 (murine macrophage) and C6 (rat astrocytic glioma) cells.

## 2. Results and Discussion

Fresh thawed tissues of both *A. elegans* and *C. edwardsii*, collected in the bay of Naples, immediately frozen, and kept at −28 °C until extraction, were exhaustively extracted with methanol and, subsequently, with chloroform. For each ascidian sample, the extracts were combined and concentrated; the resulting aqueous suspensions were then partitioned between water and butanol. The butanol extract of *A. elegans* was fractionated by MPLC over a reversed phase C-18 column using stepwise elution with aqueous MeOH. The fractions eluted with 50% aqueous MeOH were further separated and purified by reversed-phase HPLC eluting with 70% aqueous MeOH containing 0.1% TFA, thus affording compounds **1** and **2** in the pure state. The butanol extract of *C. edwardsii* was fractionated by reversed-phase MPLC under the same conditions as described for that of *A. elegans*. Further separation and purification of both fractions eluted with 70% and 50% aqueous MeOH by repeated reversed-phase HPLC yielded compounds **3**–**5** in the pure state.

A first survey of the 1D NMR spectra (CD_3_OD) of **1** and the comparison with those of the already known compound **4** readily allowed us to hypothesize that **1** was the 15-hydroxy analogous of **4**. The proton spectrum of **1** lacked indeed the signals due to the isopropyl terminus portion of **4**, whereas it contained a quite deshielded methyl signal (δ 1.14), resonating as a singlet and integrating for six protons. Likewise, the ^13^C-NMR spectrum of **1** contained a quaternary carbon resonance (δ 70.8) attributable to an oxygen-bearing carbon while the methine signal at δ 29.1 due to C-15 in **4** was absent. Chemical shifts and coupling patterns of the remaining signals of **1**, assigned by aid of COSY, HSQC, and HMBC experiments were very similar to those of **4**. Mass data analysis confirmed the assumption made. ESI mass spectrum (negative ion mode) contained an ion peak at *m*/*z* 511; an intense fragment at *m/z* 453 was present and it was interpreted as the result of an α cleavage at C-15. High resolution analysis on the pseudomolecular ion peak gave *m/z* 511.1987, which was consistent with the molecular formula C_20_H_40_NaO_9_S_2_ corresponding to [M−Na]^−^ (calculated value: 511.2006). The location of the hydroxyl group at C-15 was unambiguously established through 2D-NMR experiments which allowed us also to assign all the proton and carbon resonances of compound **1** (Table 1). In particular, diagnostic ^3^*J*_C-H_ long-range couplings were observed in the HMBC spectrum between the signal at δ 1.14 (s, 6H, CH_3_-16/20) and both the oxygenated quaternary carbon at δ 70.8 (C-15) and the methylene carbon at δ 44.4 (C-14). 

**Table 1:** ^1^H and ^13^C-NMR data of compounds **1**–**3** in CD_3_OD.

	1		2		3	
Pos.	δ_H_ (mult., *J* in Hz)	δ_C_	δ_H_ (mult., *J* in H*z*)	δ_C_	δ_H_ (mult., *J* in H*z*)	δ_C_
**1**	4.02 (m)	66.7	3.80 (H_a_, dd, 6.6, 9.1)	73.2	3.79 (H_a_, dd, 6.6–9.4)	73.6
			3.88 (H_b_, dd, 6.6, 9.1)		3.87 (H_a_, dd, 6.6–9.4)	
**2**	1.42 ^a^ (H_a_)	36.7	1.78 (m)	33.5	1.79 (m)	34.1
	1.68					
	1.68 (m, H_b_)					
**3**	1.60 (m)	29.9	1.14 (m)	33.7	1.14 (m)	34.3
			1.40		1.43	
			1.41 ^a^		1.4 ^a^	
**4**	1.10 ^a^	37.6	1.33 ^a^	24.5	1.32 ^a^	25.1
	1.30					
	1.30 ^a^					
**5**	1.30 ^a^	24.6	1.10 ^a^	37.6	1.11 ^a^	38.1
			1.30		1.32	
			1.30 ^a^		1.30 ^a^	
**6**	1.10 ^a^ (H_a_)	37.6	1.39 ^a^	33.0	1.41 ^a^	33.5
	1.30					
	1.28 ^a^ (H_b_)					
**7**	1.40 ^a^	33.1	1.10 ^a^	37.6	1.11 ^a^	38.1
			1.30		1.32	
			1.30 ^a^		1.30 ^a^	
**8**	1.09 ^a^ (H_a_)	37.7	1.33 ^a^	24.5	1.32 ^a^	25.1
	1.28					
	1.28 ^a^ (H_b_)					
**9**	1.29 ^a^	24.6	1.31 ^a^	31.7	1.23 ^a^	31.9
			1.36		1.33	
			1.36 ^a^		1.32 ^a^	
**10**	1.30 ^a^	32.0	1.66 (m)	38.4	1.44 (m)	41.4
	1.38					
	1.38 ^a^					
**11**	1.65 (m)	38.4	1.30 ^a^	31.9	1.23 ^a^	31.9
			1.38		1.33	
			1.38 ^a^		1.32 ^a^	
**12**	1.36 ^a^	31.7	1.33 ^a^	24.5	1.33 ^a^	25.2
**13**	1.30 ^a^	24.6	1.42 ^a^	44.4	1.19 (m)	40.3
**14**	1.42 ^a^	44.4	-	70.7	1.55 (m)	28.9
**15**	-	70.8	1.15 (s)	28.3	0.89 (d, 6.4)	22.8
**16**	1.14 (s)	28.4	0.94 (d, 6.6)	16.3	0.96 (d, 6.6)	16.9
**17**	0.90 (d, 6.6)	18.9	0.86 (d, 6.6)	19.2	0.88 (d, 6.4)	19.9
**18**	0.85 (d, 6.6)	19.2	3.92 (d, 5.4)	70.8	3.44 (d, 5.4)	65.5
**19**	3.91 (d, 5.4)	70.7	1.15 (s)	28.3	0.89 (d, 6.4)	22.8
**20**	1.14 (s)	28.4	-	-	-	-
					3.86	

^a^ Signals overlapped by other resonances.

The negative HRESI mass spectrum (negative ions) of compound **2** displayed an ion peak at *m/z* 497.1825 corresponding to [M−Na]^−^ (calculated value: *m/z* 497.1849); the molecular formula of **2** was thus established as C_19_H_38_NaO_9_S_2_. The ^1^H- and ^13^C-NMR spectra of compound **2** displayed a close resemblance to those of **1**, and the observed differences were due to the initial segment of the linear skeleton of both compounds (C1-C3/C16 in **2** and C1-C4/C17 in **1**). The multiplet at δ 4.02 due to 2H-1 in compound **1** was replaced by an ABX system at δ 3.80 (1H, dd, *J* = 9.1 and 6.6 Hz, H-1a) and 3.86 (1H, dd, *J* = 9.1 and 6.6 Hz, H-1b). COSY connectivities, which allowed to delineate the large spin system C1-C13 through CH_3_-16, CH_3_-17 and CH_2_-18, as well as HMBC information, clearly evidenced **2** as the 1-nor-derivative of **1** or, alternatively, the 15-hydroxy analogue of **5** (Table 1).

The NMR features (CD_3_OD) of compound **3** appeared almost identical to those of **5**, except for the chemical shift of the C-18 methylene protons as well as that of the relevant carbon, both showing a significant upfield shift (δ_H_: 3.94 in **5***vs*. 3.44 in **3**; δ_C_: 73.9 in **5** *vs*. 65.5 in **3**). The negative ion HRESI mass spectrum of **3** displayed an ion peak at *m/z* 379.2522 which was consistent with the molecular formula C_19_H_39_O_5_S^−^, corresponding to [M−Na]^−^(calculated value 379.2513). These few data readily allowed us to deduce that **3** was the 18-desulfated analogue of **5**; this conclusion was fully corroborated by 2D NMR spectra analysis which also led to the full assignment of all NMR resonances in **3** (Table 1).

**Figure 2:**
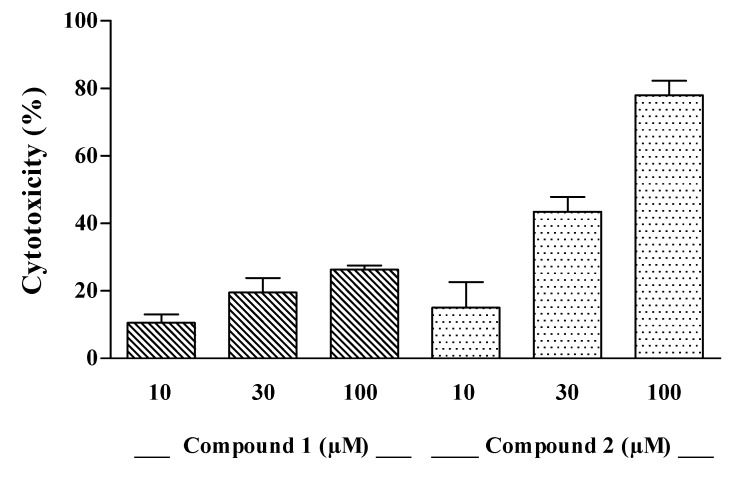
Cytotoxic effect of compounds **1** and **2** at increasing concentrations on J774A.1 cells. Each per cent value is the mean ± SEM of three independent experiments.

The cytotoxic effect of compounds **1**–**3** was evaluated on J774A.1 (BALB/c murine macrophages) and C6 (rat glioma) cell lines *in vitro*. Compounds **1** and **2** induced a concentration-dependent mortality on J774A.1 (Figure 2) whereas both drugs were inactive on C6 cells, with a LC_50_ > of 300 µM (data not shown). When comparing optical density values of control and treated cells, the cytotoxic effect of compound **1** and **2** was significant at the highest concentrations (*p* < 0.05, compound **1** at 100 µM; *p* < 0.05 and *p* < 0.01, compound **2** at 30 and 100 µM, respectively). The LC_50_ value of compound **2** on J774A.1 cell line was 45.12 µM, while that of compound **1** was > of 100 µM (Figure 2). The compound **3** was ineffective on both J774A.1 and C6 cells (data not shown). On the basis on these although preliminary data, a sulfate group in R2 and/or a hydroxyl group in R1 seems to be essential for the cytotoxic activity, since they are absent in compound **3** which proved inactive. Nevertheless, previous reports showed that compounds **4** and **5**, possessing exclusively the sulfate group in R2, are still active [10,14] therefore, we can conclude that only the latter functionality has an obligatory role for the cytotoxic activity showed by compounds **1** and **2** against the macrophagic cell line. The slightly different potency of compounds **1** and **2** could be ascribed to the different length chain; this hypothesis is supported by cytotoxicity data reported in literature for compounds **4** and **5**, where compound **5**, characterized by a shorter C-chain, resulted more active than its higher analogue **4** [10,14].

## 3. Experimental

### 3.1. General Procedures

ESI mass spectra were recorded on a Micromass QTOF Micro mass spectrometer in MeOH. The spectra were recorded by infusion into the ESI source using MeOH as solvent. HRESIMS (negative mode) were performed on a Thermo LTQ Orbitrap XL mass spectrometer. Optical rotations were measured with a Perkin-Elmer 192 polarimeter at 589 nm using a 10 cm microcell. ^1^H (500 MHz) and ^13^C (125 MHz) NMR spectra were recorded on a Varian INOVA spectrometer; chemical shifts were referenced to the residual solvent signal (methanol-*d_4_*: δ_H_ 3.31, δ_C_ 49.0). Homonuclear ^1^H connectivities were determined by COSY and TOCSY (mixing time 100 ms) experiments. Through-space ^1^H connectivities were evidenced using a ROESY experiment with a mixing time of 500 ms. Two and three bond ^1^H-^13^C connectivities were determined by gradient 2D HMBC experiments optimized for a J_2,3_ of 8 Hz. All spectra were acquired at 278 K, and samples were prepared by dissolving compounds **1**–**3** in 0.5 mL of methanol-*d_4_* (Armar, 100% D).

### 3.2. Extraction and Isolation

Specimens of *A.elegans* were collected at −40 m depth in the autumn of 2003 in the Bay of Naples (Bacoli) and kept frozen until used. The freshly thawed tunicate was homogenized and extracted at room temperature with methanol (3 × 1 L) and, subsequently, with chloroform (3 × 1 L) (31.8 g dry weight of the tunicate after air drying). The combined extracts were concentrated *in vacuo* to give an aqueous suspension that was subsequently partitioned initially with EtOAc and then with *n*-BuOH. The butanol-soluble material obtained after evaporation of the solvent (4.1 g of a dark brown oil), was chromatographed on a RP-18 silica gel flash column using a gradient elution (H_2_O/MeOH 9:1→ H_2_O/MeOH 7:3→ H_2_O/MeOH 1:1→ H_2_O/MeOH 3:7→ MeOH 100%→ MeOH/CHCl_3_ 9:1→ MeOH/CHCl_3_ 7:3→ MeOH/CHCl_3_ 1:1→ CHCl_3_ 100%). The fractions eluted with H_2_O/MeOH 1:1 were rechromatographed by HPLC on an RP-18 column (Luna, 3 μm, 150 × 4.60 mm), using H_2_O/MeOH 7:3 containing TFA 0.1% as the eluent (flow 0.5 mL/min). This separation afforded pure compound **1** (1.2 mg) and pure compound **2** (1.0 mg).

Specimens of *C.* *edwardsii* were collected at −75 m depth in the autumn of 2006 in the Bay of Naples (at −65/−75 meter in Meta di Sorrento Punta Gradelle) and kept frozen until used. The freshly thawed tunicate was homogenized and treated at room temperature with methanol (3 × 1 L) and, subsequently, with chloroform (3 × 1 L) (21.9 g dry weight of the tunicate after air drying). The combined extracts were concentrated *in vacuo* to give an aqueous suspension that was subsequently partitioned initially with EtOAc and then with *n*-BuOH. The butanol-soluble material obtained after evaporation of the solvent (5.4 g of a dark brown oil), was chromatographed on a RP-18 silica gel flash column using a gradient elution (H_2_O/MeOH 9:1→ H_2_O/MeOH 7:3→ H_2_O/MeOH 1:1→ H_2_O/MeOH 3:7→ MeOH 100%→ MeOH/CHCl_3_ 9:1→ MeOH/CHCl_3_ 7:3→ MeOH/CHCl_3_ 1:1→ CHCl_3_ 100%). The fractions eluted with H_2_O/MeOH 3:7 were rechromatographed by HPLC on an RP-18 column (Luna, 3 μm, 150 × 4.60 mm), using H_2_O/MeOH 72:28 containing TFA 0.1% as the eluent (flow 0.5 mL/min). This separation afforded 2.4 mg of pure compound **3**.

### 3.3. Compound ***1***

Colorless amorphous solid; [*α*]_D_^25^ = +2.5 (*c* = 0.002, CH_3_OH). ESI-MS (negative ion mode): *m/z* = 511 [M−Na]^−^; HRESIMS (negative ion mode): *m/z* = 511.1987; the molecular formula C_20_H_40_NaO_9_S_2_^−^ requires 511.2006; ^1^H-NMR and ^13^C-NMR data (CD_3_OD, 500/125 MHz) are reported in Table 1.

### 3.4. Compound ***2***

Colorless amorphous solid; [*α*]_D_^25^ = +3.4 (*c* = 0.002, CH_3_OH). ESI-MS (negative ion mode): *m/z* = 497 [M−Na]^−^; HRESIMS (negative ion mode): *m/z* = 497.1825; the molecular formula C_19_H_38_NaO_9_S_2_^−^ requires 497.1849; ^1^H-NMR and ^13^C-NMR data (CD_3_OD, 500/125 MHz) are reported in Table 1.

### 3.5. Compound ***3***

Colorless amorphous solid; [*α*]_D_^25^ = +1.8 (*c* = 0.002, CH_3_OH). ESI-MS (negative ion mode): *m/z* = 379 [M−Na]^−^; ESI-MS (positive ion mode): *m/z* = 403 [M+H]^+^; HRESIMS (negative ion mode): *m/z* = 379.2522; the molecular formula C_19_H_39_O_5_S^−^ requires 379.2513; ^1^H-NMR and ^13^C-NMR data (CD_3_OD, 500/125 MHz) are reported in Table 1.

### 3.6. Cell Culture

J774A.1 cell line (BALB/c murine macrophages) was cultured in Dulbecco’s modified Eagle’s medium (DMEM) supplemented with 4.4% NaHCO_3_ (HyClone), penicillin (100 U/mL), streptomycin (100 μg/mL), 2 mM glutamine, 25 mM Hepes, 130 µg/mL Na pyruvate and 10% foetal calf serum (FCS) (Hy Clone). The rat astrocytic glioma C6 cell line was cultured in DMEM supplemented with 10% FCS, penicillin (100 U/mL), streptomycin (100 μg/mL), and L-glutamine 2 mM. All cells were cultured in plastic tissue culture flasks and kept at 37 °C under 5% CO_2_ atmosphere.

### 3.7. Cytotoxicity Assay

Cytotoxicity studies in both tumour cell lines were performed in a 96-well plate [17,18]. J774A.1 were mechanically scraped, while C6 cells were enzymatically detached. The cells were plated 12.5 × 10^4^/well (J774A.14) or 2.5 × 10^3^/well (C6) to a final volume of 200 μL. After 4 h J774A.1, cells were incubated with compounds **1**–**3** at increasing concentrations (10–100 μM) for 22 h; conversely, after 3 days C6 cells were incubated with compounds **1**–**3** (10–300 µM) for 22 h. Then, 25 μL of 3-(4,5-dimethylthiazol-2-yl)-2,5-diphenyltetrazolium bromide (MTT, 5 mg/mL) was added in each well, 3 h later the cells were lysated with 100 μL of lysis buffer (20% SDS and 50% DMF, pH 4.7). After an incubation of 22 h at 37 °C the optical densities (OD_620_) for the serial dilutions of both compounds were compared with the OD of the control wells to assess the citotoxicity [19]. LC_50_ for each cell line was obtained by statistical computer program.

### 3.8. Data Analysis

Data are reported as mean ± SEM values of three independent determinations. All experiments were performed at least three times, each time with three or more independent observations. Statistical analysis was performed by ANOVA test, and multiple comparisons were made by Dunnett test.

## 4. Conclusions

The structures of compounds **1**–**3**, isolated from the Mediterranean ascidians *Aplidium elegans* and *Ciona edwardsii*, have been elucidated using mass spectrometry and NMR experiments and their *in vitro* cytotoxic effects have been evaluated on J774A.1 (BALB/c murine macrophages) and C6 (rat glioma) cell lines. A moderate but selective cytotoxic effect on J774A.1 cell line has been evidenced for compounds **1** and **2**; the inactivity of **3**, as well as further pharmacological data available in the literature [10,14], indicated that the hydroxyl group does not confer *per se* cytotoxic activity, while the contribution of the sulfate is of pivotal importance. A slight influence of alkyl chain length on the potency of the active compounds has been also evidenced. 

## Supplementary Materials

Supplementary materials can be accessed at: http://www.mdpi.com/1420-3049/17/11/12642/s1.
