# Supplementary material for: New Bioactive Alkyl Sulfates from Mediterranean Tunicates
Source: Molecules. 2012 Oct 26;17(11):12642–50. doi: 10.3390/molecules171112642 (PMC6268736; doi:10.3390/molecules171112642)

Supplementary Materials

^{1}H-NMR spectrum of compound **1**	S2
COSY spectrum of compound **1**	S3
HSQC spectrum of compound **1**	S4
HMBC spectrum of compound **1**	S5
HRESI mass spectrum of compound **1**	S6
^{1}H-NMR spectrum of compound **2**	S7
COSY spectrum of compound **2**	S8
HSQC spectrum of compound **2**	S9
HMBC spectrum of compound **2**	S10
HRESI mass spectrum of compound **2**	S11
^{1}H-NMR spectrum of compound **3**	S12
COSY spectrum of compound **3**	S13
HSQC spectrum of compound **3**	S14
HMBC spectrum of compound **3**	S15
HRESI mass spectrum of compound **3**	S16

^{1}H-NMR spectrum of compound **1**.

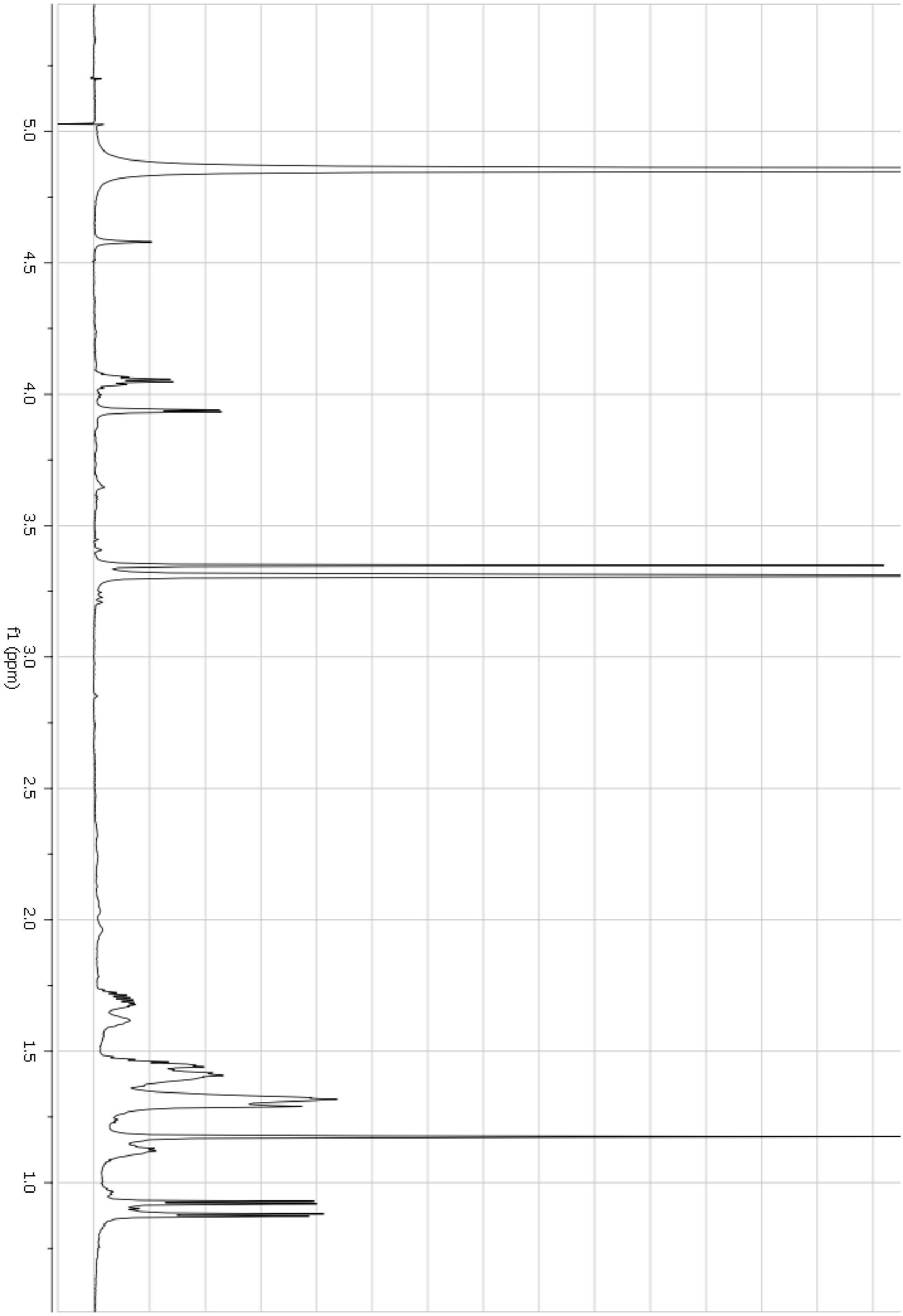

COSY spectrum of compound **1**.

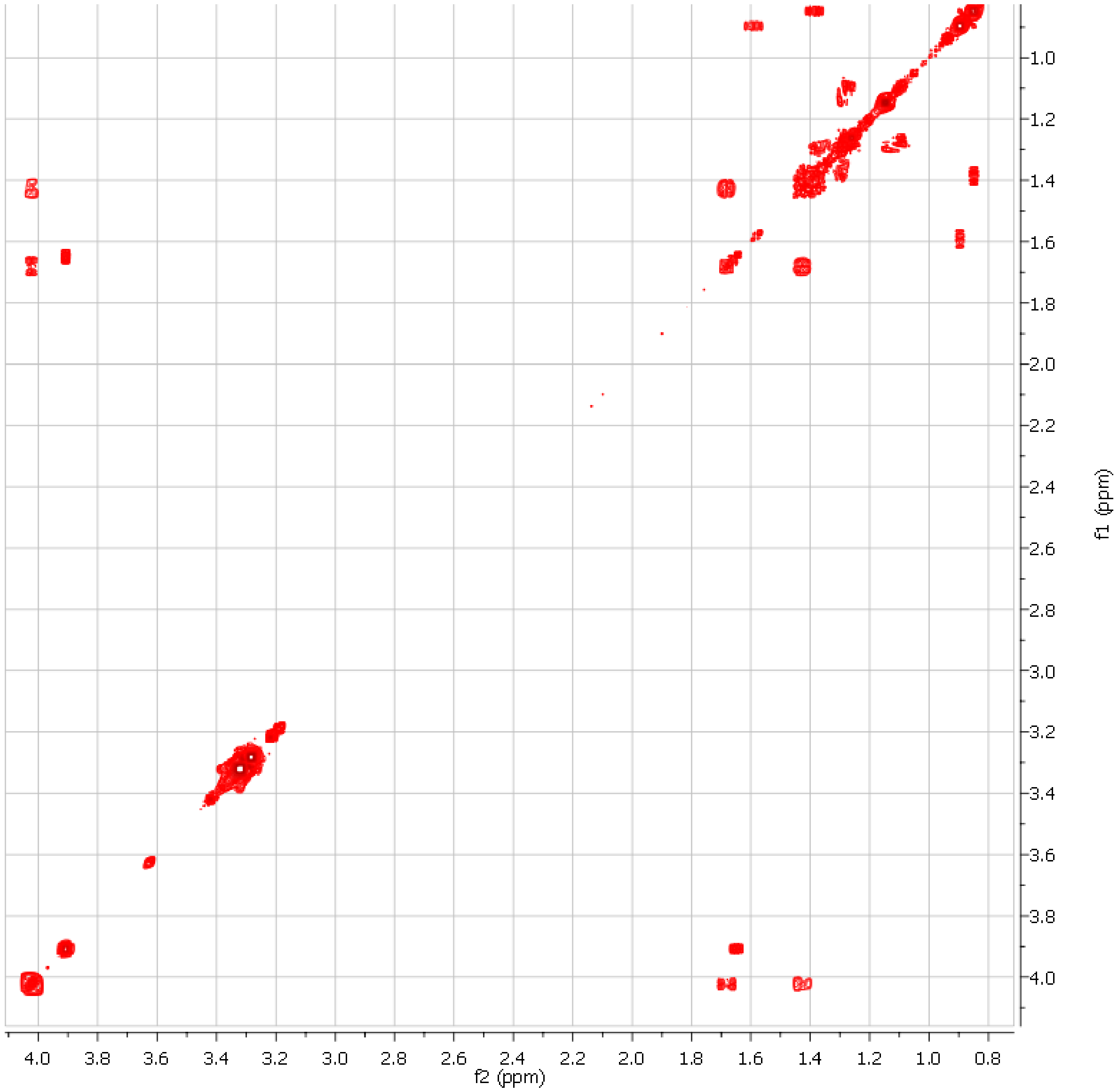

HSQC spectrum of compound **1**.

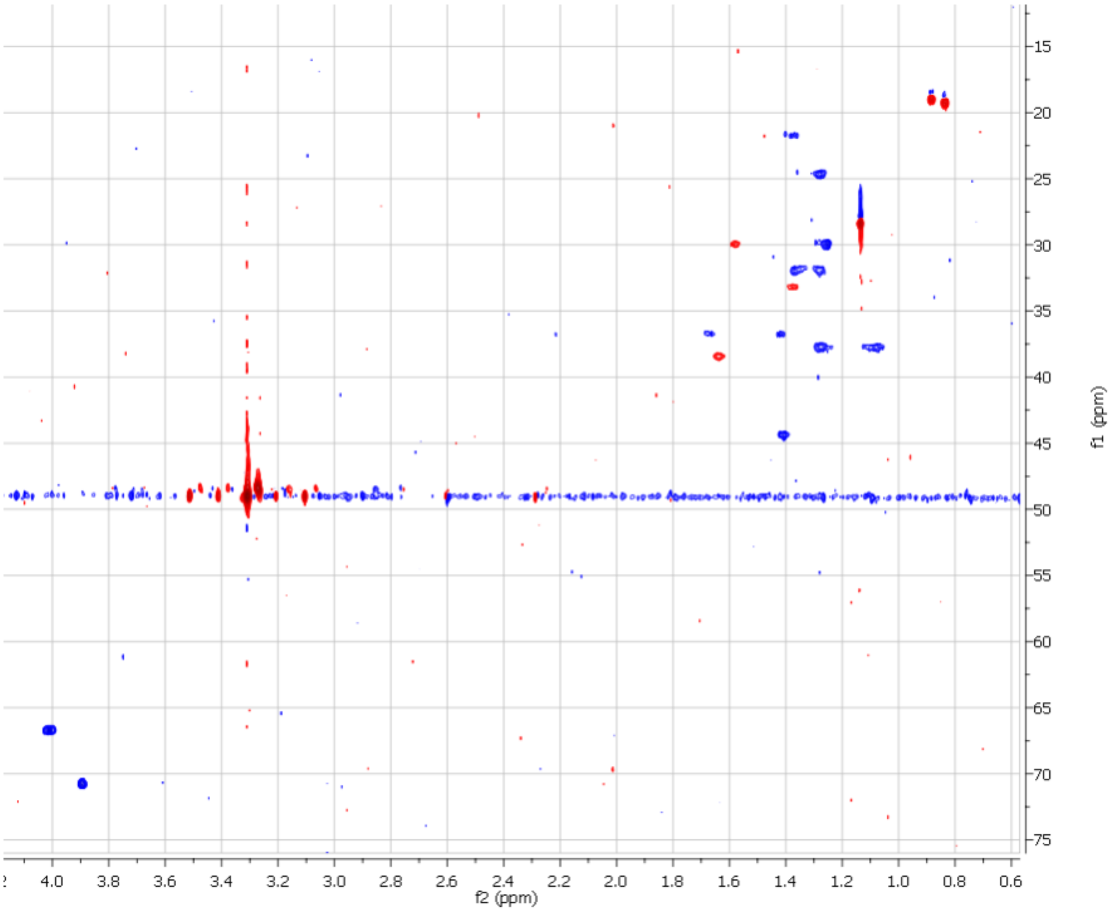

HMBC spectrum of compound **1**.

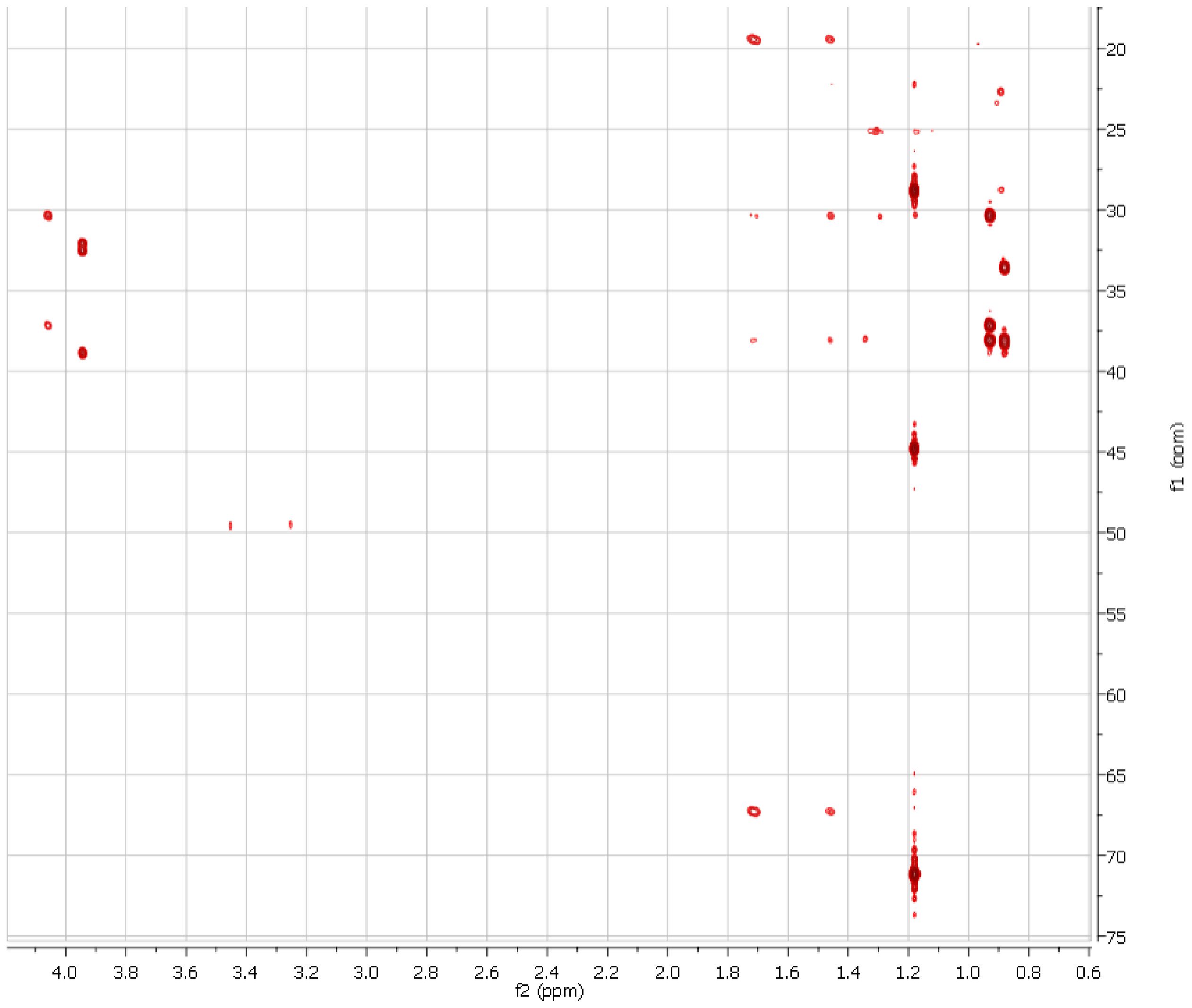

HRESI mass spectrum of compound **1**.

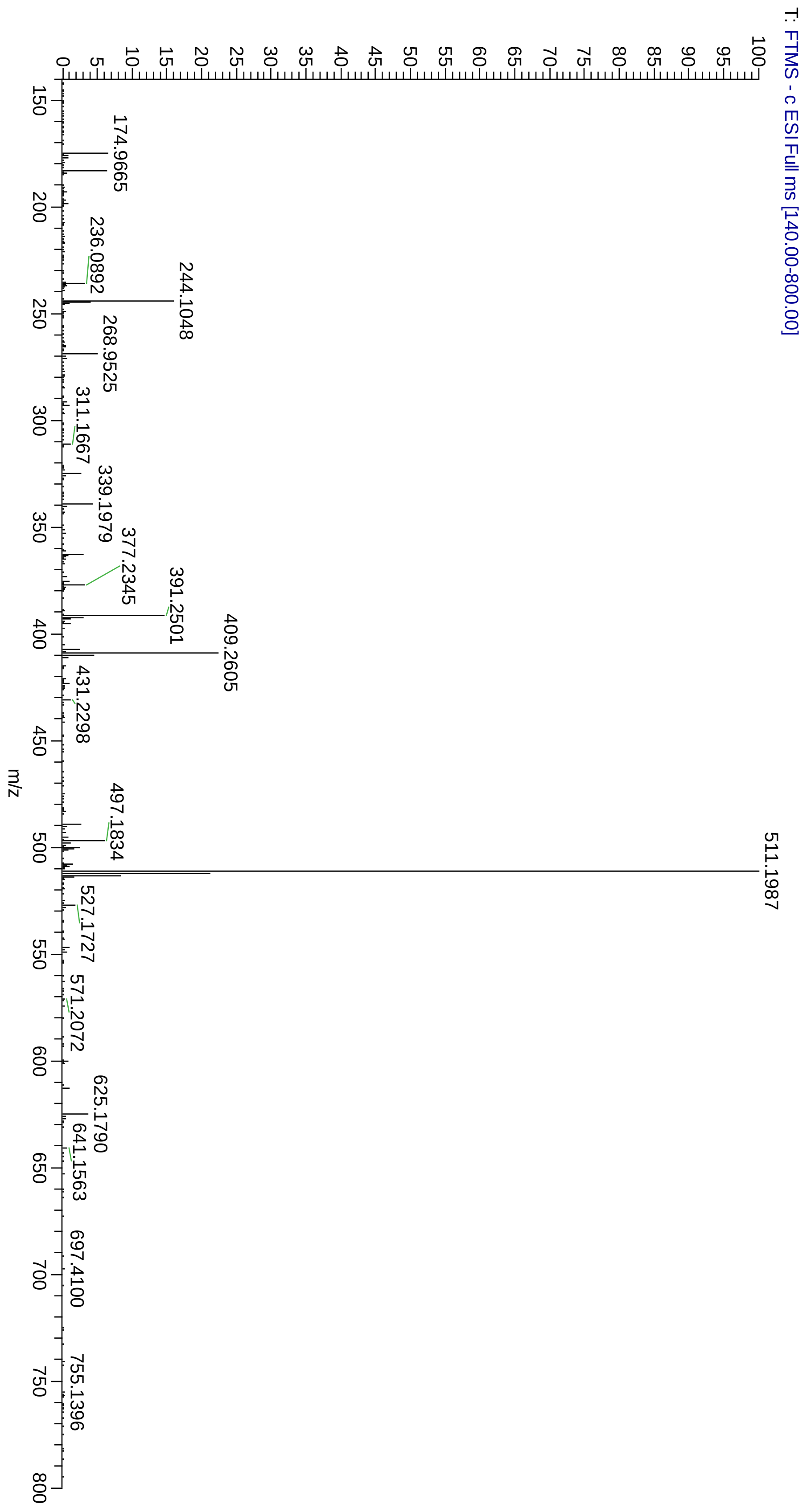

^{1}H-NMR spectrum of compound **2**.

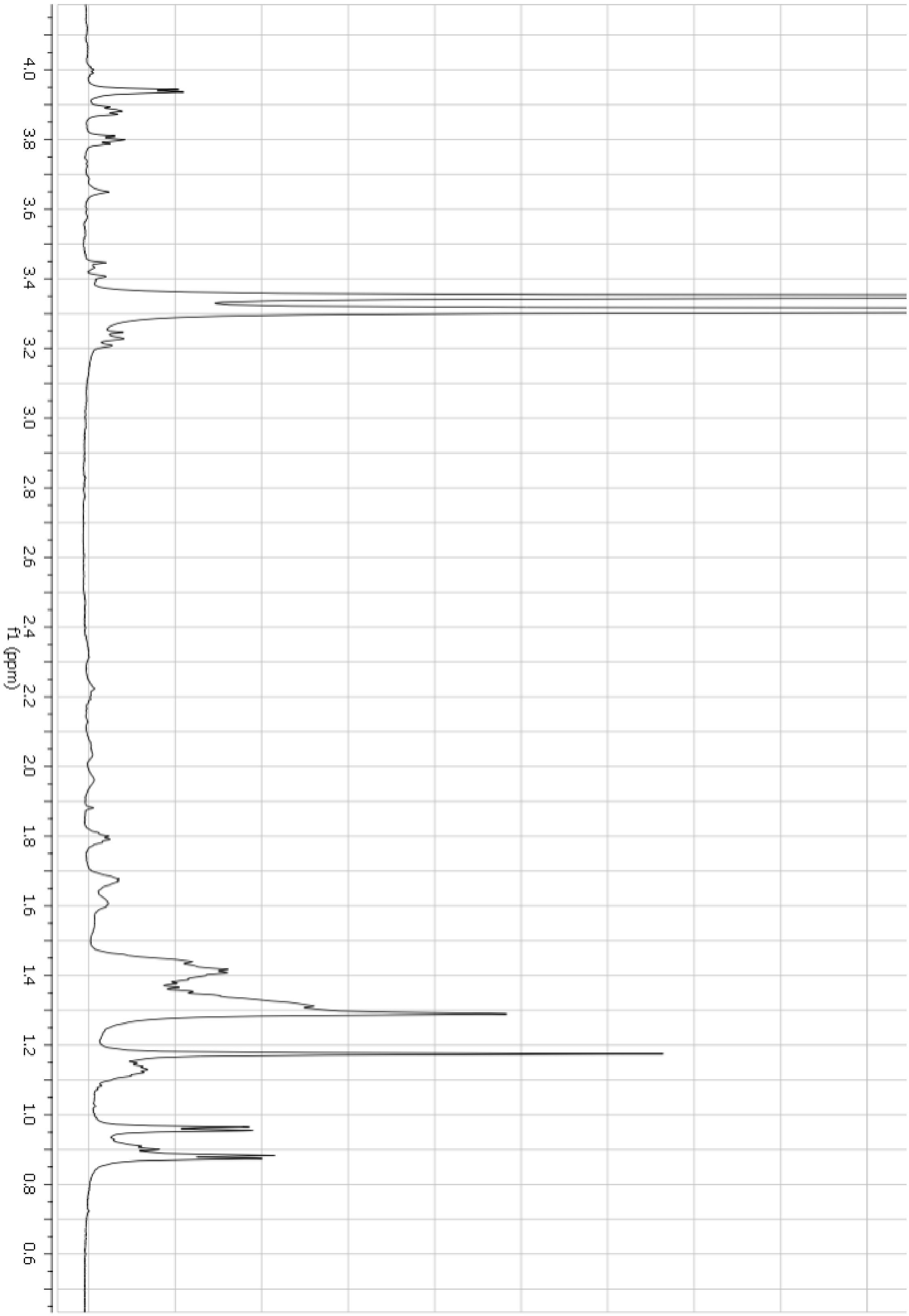

COSY spectrum of compound **2**.

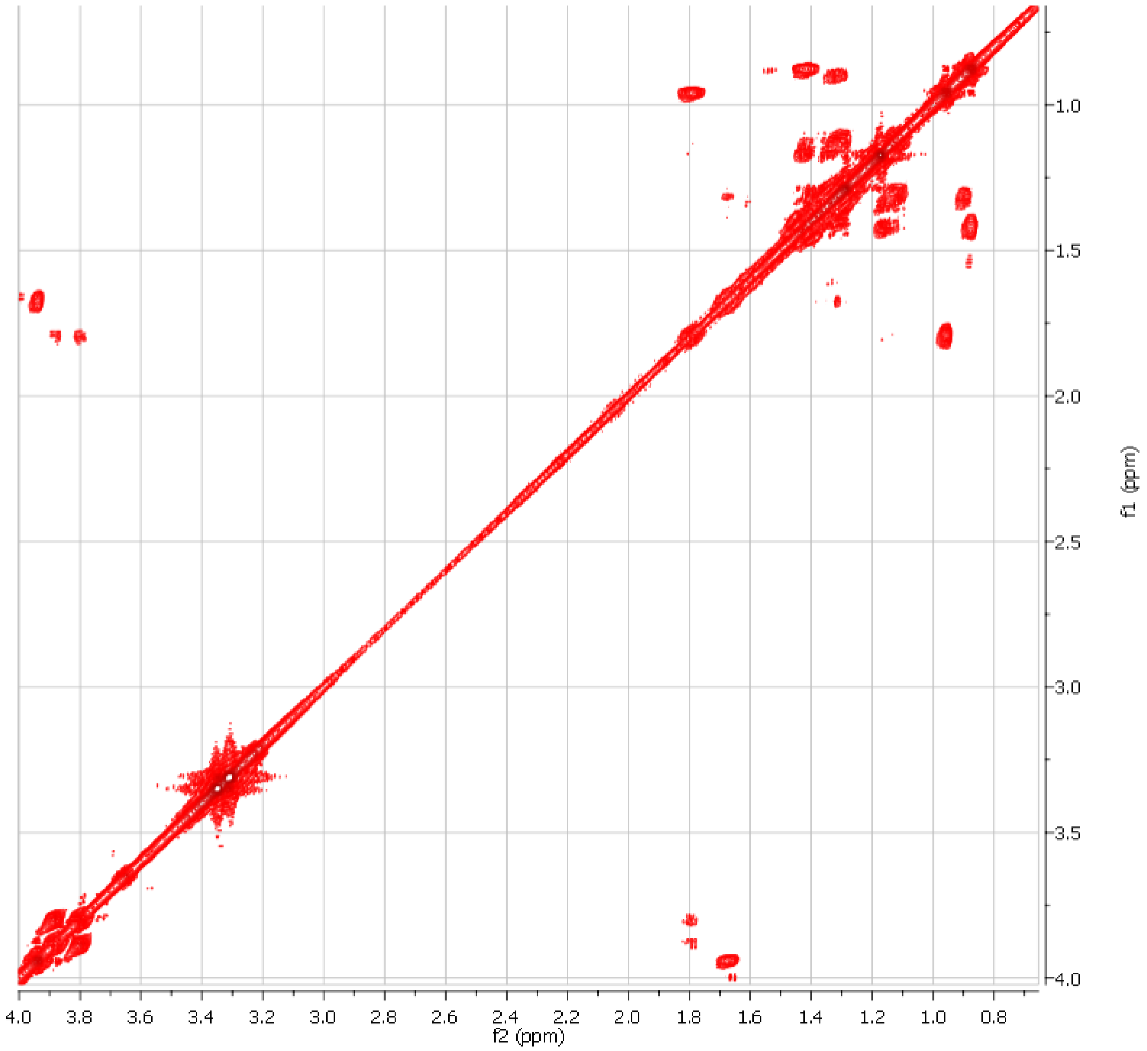

HSQC spectrum of compound **2**.

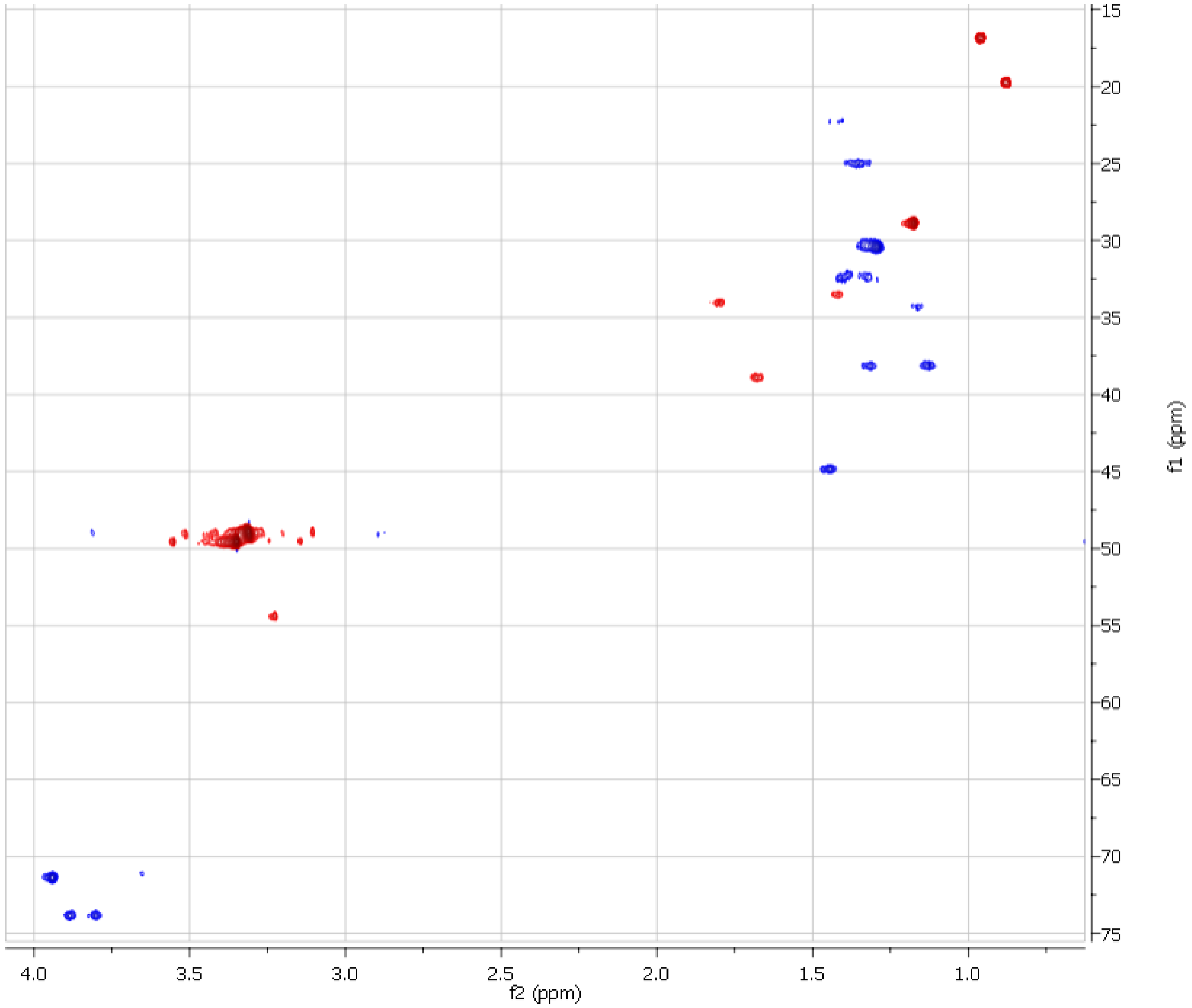

HMBC spectrum of compound **2**.

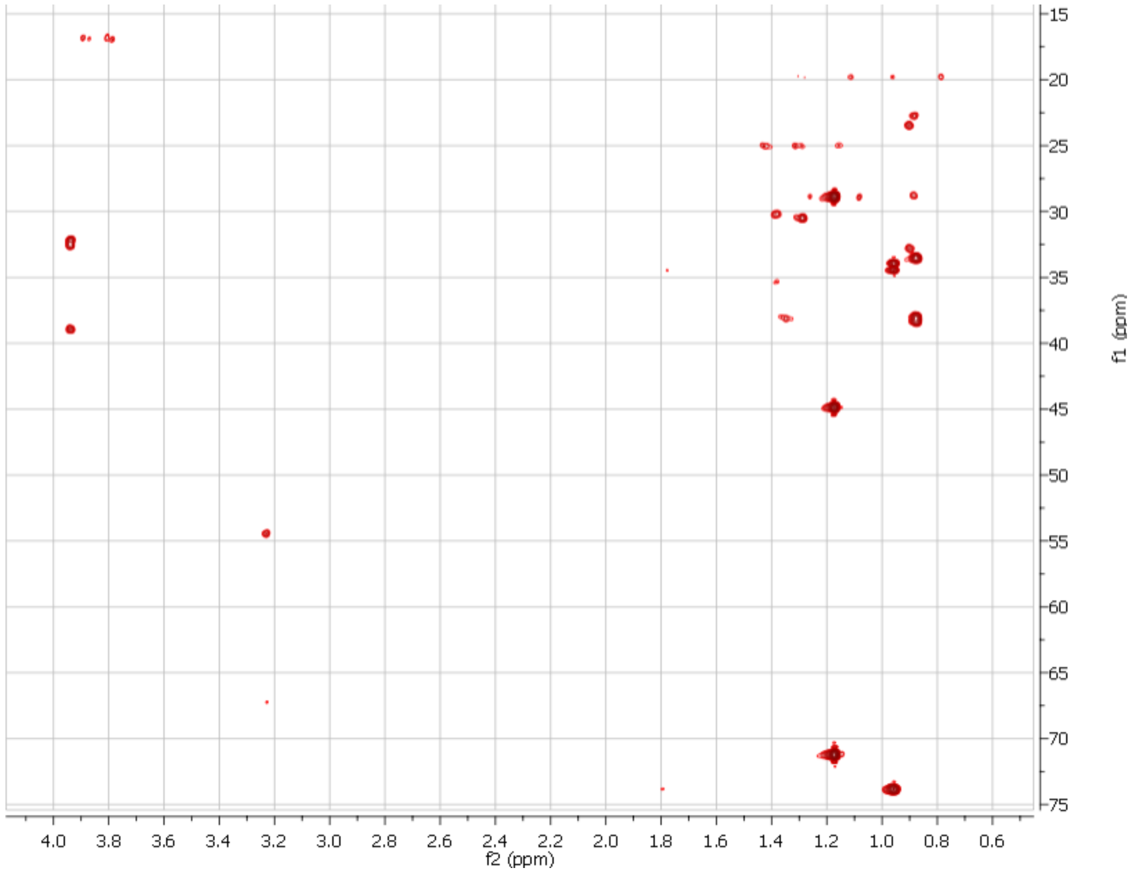

HRESI mass spectrum of compound **2**.

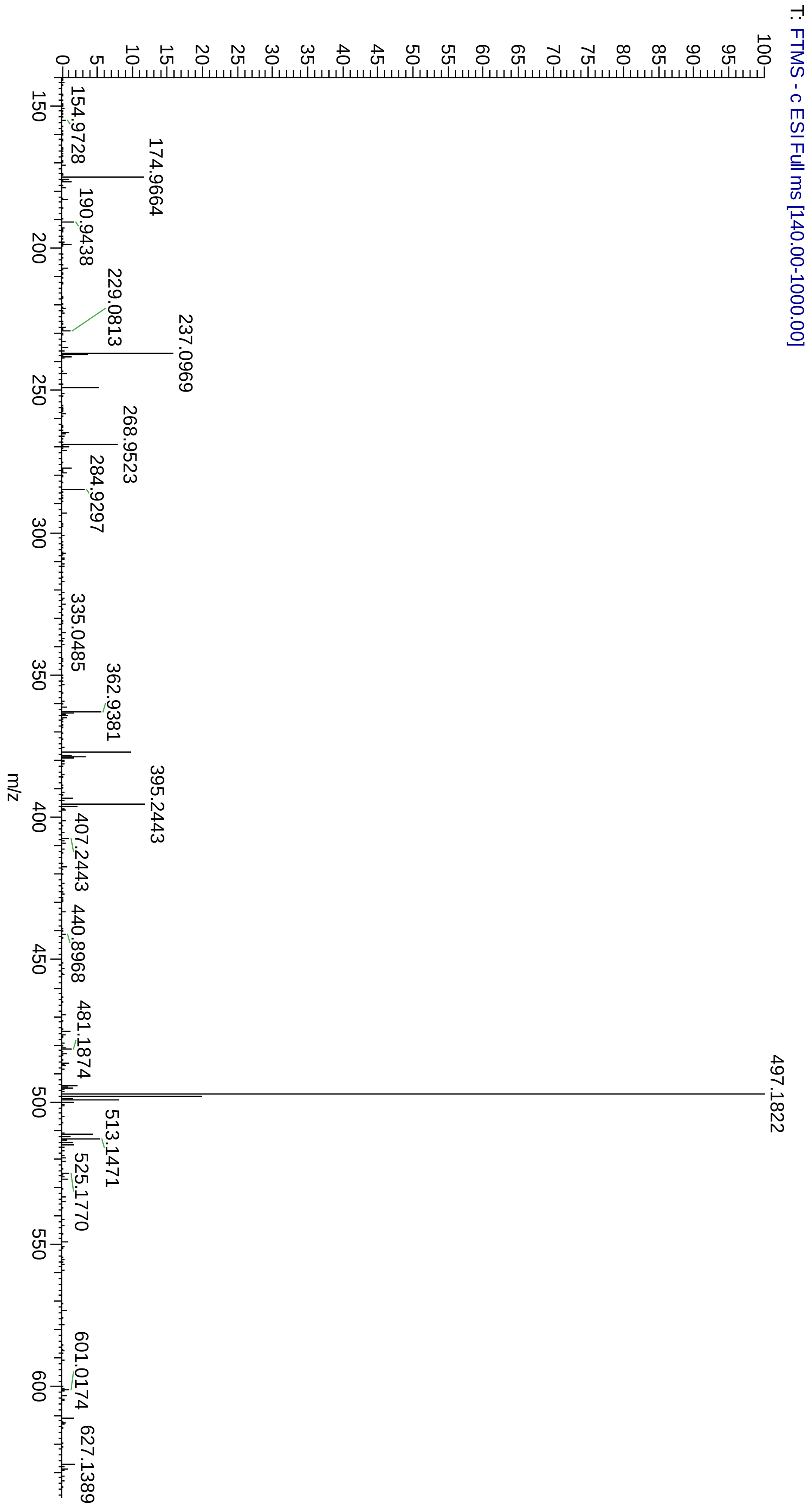

^{1}H-NMR spectrum of compound **3**.

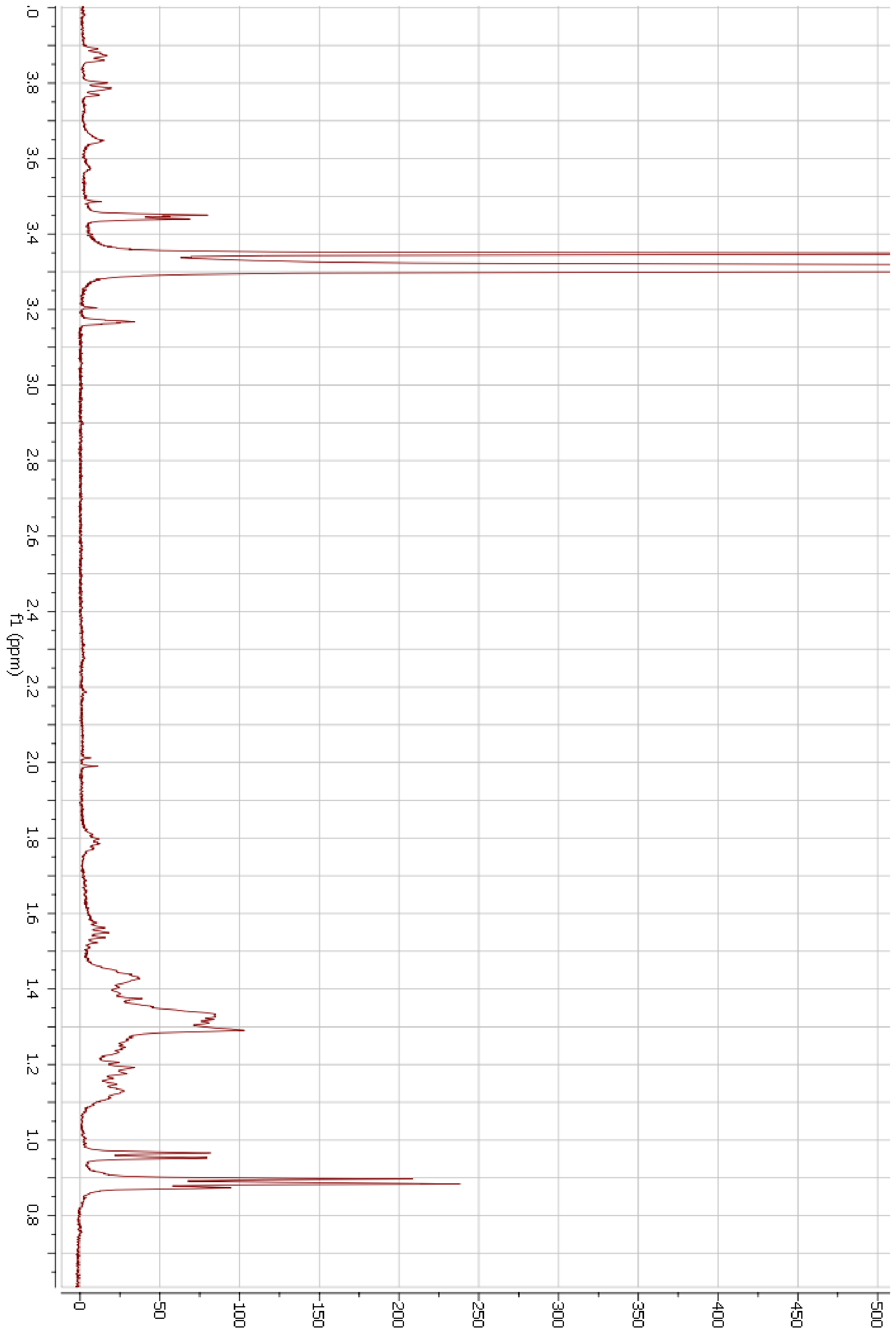

COSY spectrum of compound **3**.

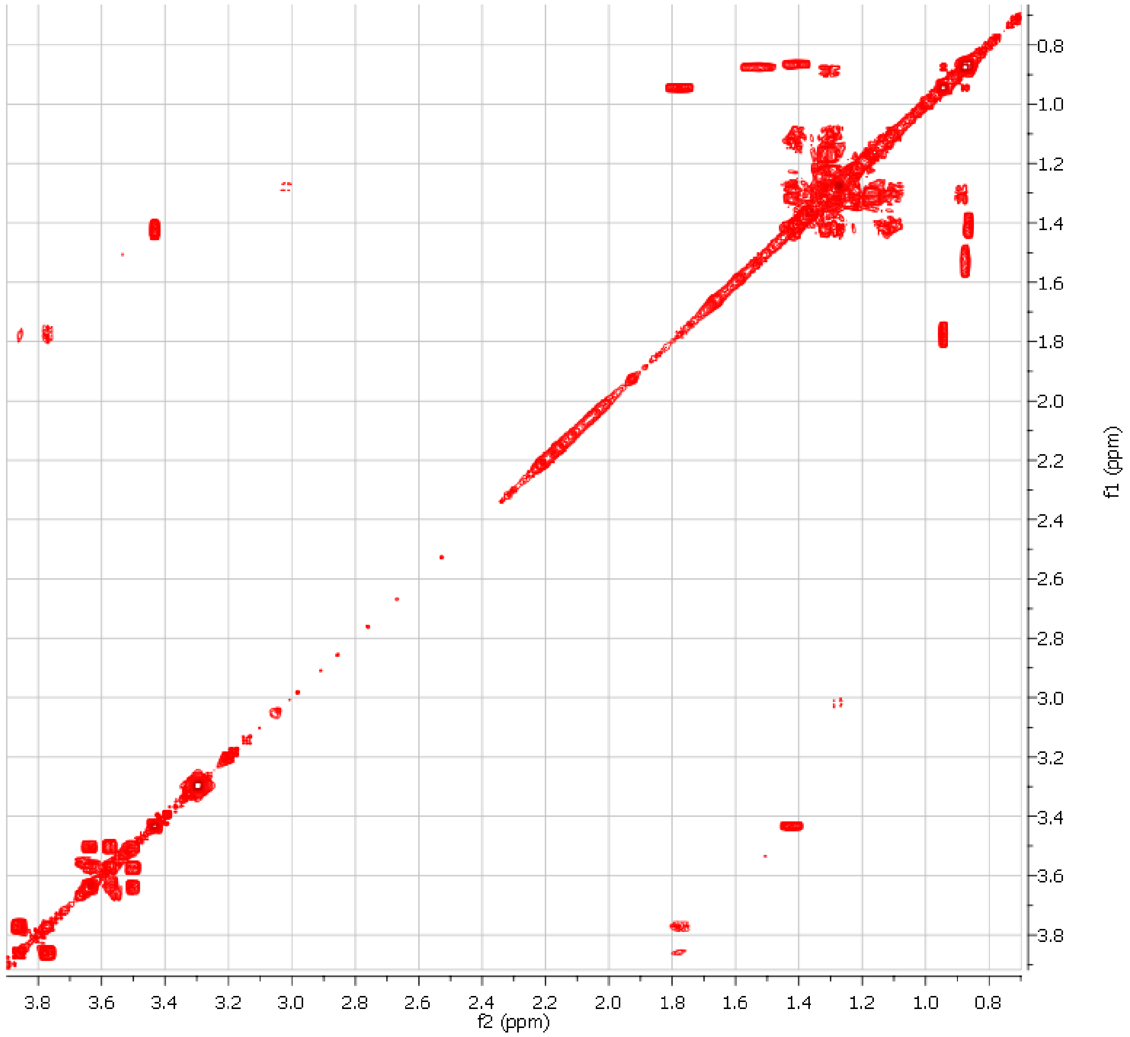

HSQC spectrum of compound **3**.

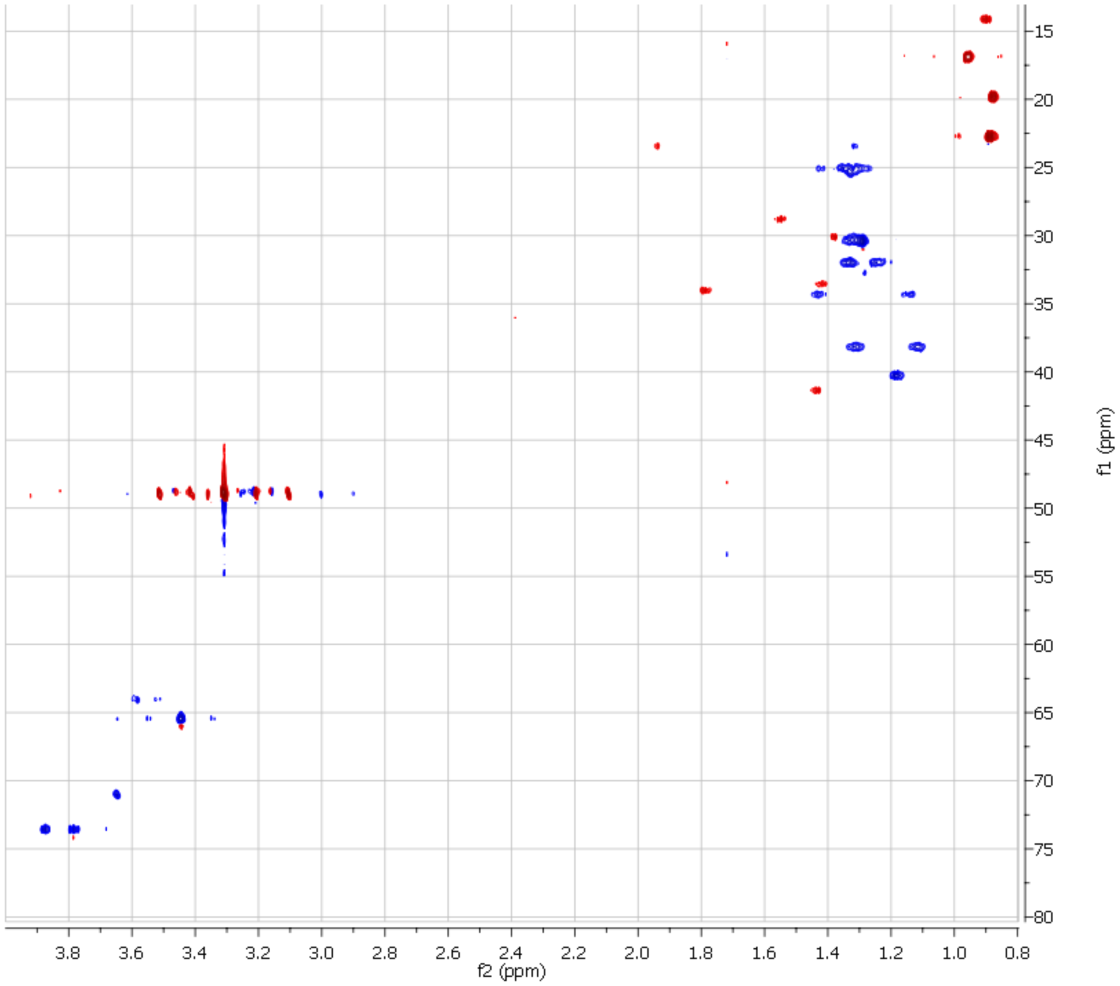

HMBC spectrum of compound **3**.

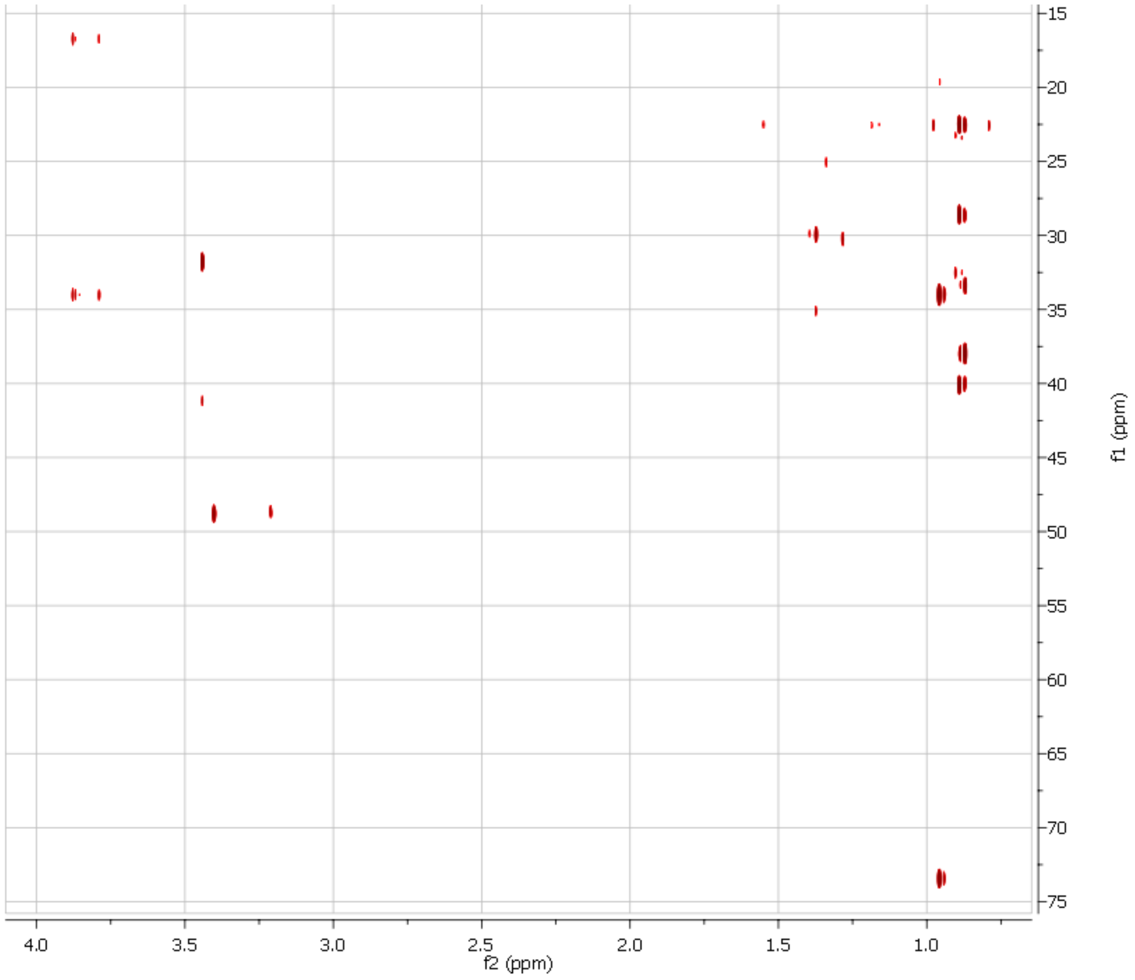

HRESI mass spectrum of compound **3**.

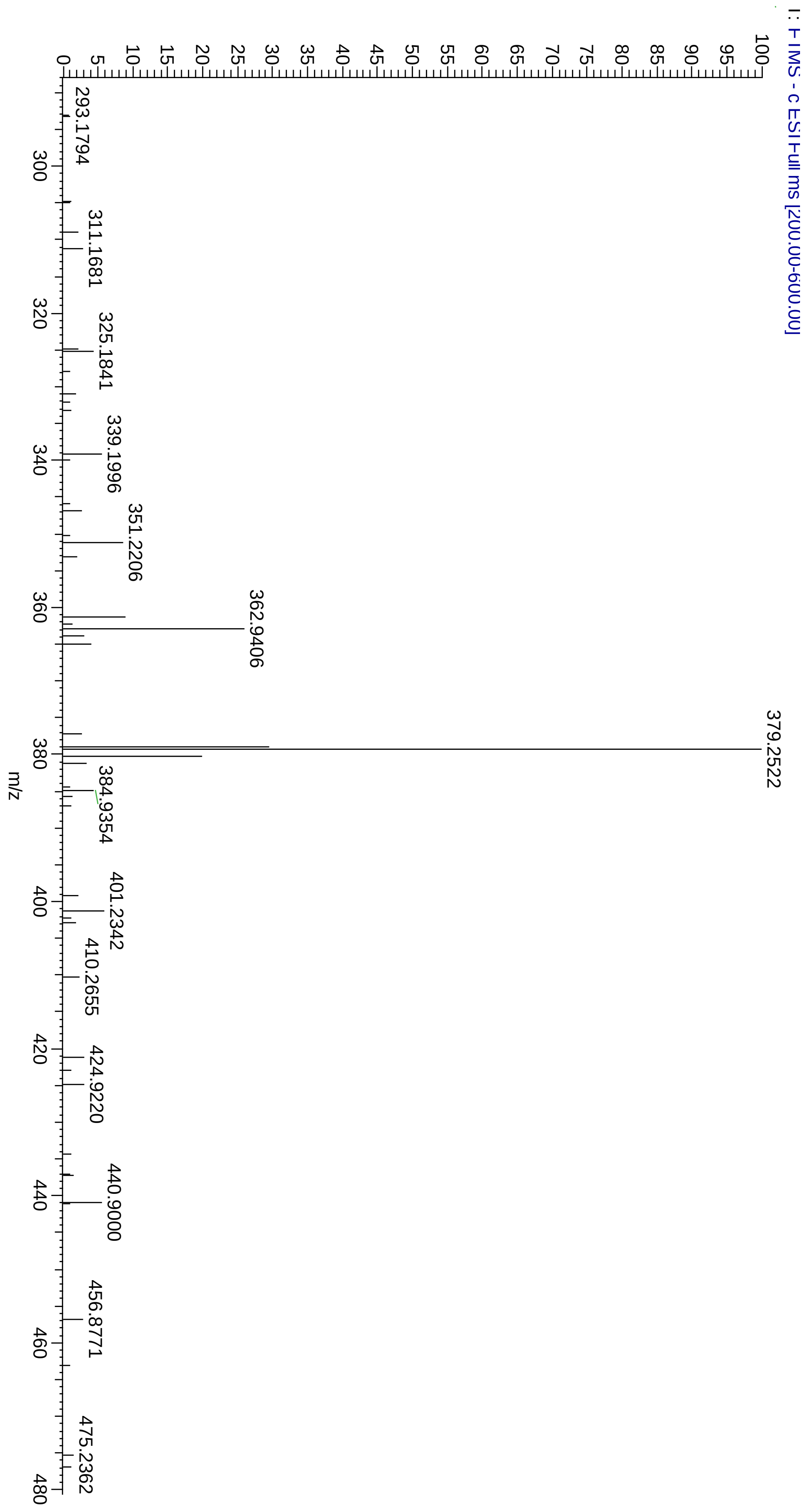

Supplement: Supplementary file 1 [file molecules-17-12642-s001.pdf]
